# Tips and Tricks in the Laparoscopic Treatment of Type I Duodenal Atresia: Description of a Technique

**DOI:** 10.3390/children12040517

**Published:** 2025-04-17

**Authors:** Salvatore Fabio Chiarenza, Maria Luisa Conighi, Valeria Bucci, Cosimo Bleve

**Affiliations:** Pediatric Surgery Unit, Regional Center of Pediatric Urology and Minimally Invasive Surgery and New Technologies, AULSS 8, S. Bortolo Hospital, 36100 Vicenza, Italy; fabio.chiarenza@aulss8.veneto.it (S.F.C.); marialuisa.conighi@aulss8.veneto.it (M.L.C.); valeria.bucci@aulss8.veneto.it (V.B.)

**Keywords:** duodenal atresia, duodenal web, late presentation, atresia, laparoscopy, endoscopy, indocyanine green

## Abstract

Introduction: Congenital duodenal atresia (DA) (Type I) with a fenestrated web can be characterized by a late presentation with a delayed diagnosis. It is even rarer and usually associated with proximal duodenomegaly. Conventional management involves web resection and duodeno–duodeno anastomosis with or without duodenoplasty. We describe our mininvasive surgical strategy and management, detailing the aspects of laparoscopic techniques. Material and Methods: We retrospectively reviewed the medical records of five patients affected by fenestrated duodenal web (DA) with a delayed onset of symptoms and diagnosis who were managed in our Department over a period of 10 years (2013–2023). We analyzed the age of patients at diagnosis, clinical signs and symptoms, associated congenital anomalies, radiological and intraoperative findings, surgical treatment, and outcomes. Diagnostic examinations included ultrasound (US), Upper-Gastrointestinal Study (UGI), and Esophagogastroduodenoscopy (EGDS). Results: Three boys and two girls, median age of 5.5 months (range 3–11 months), were included in this study. Three underwent previous surgery for long-gap esophageal atresia (EA), two of Type A, and one of Type C, requiring a gastrostomy immediately after birth (delayed esophageal repair for prematurity in Type C) and subsequent delayed primary anastomosis. Major associated anomalies were EA (3), anterior ectopic anus (1), cloaca (1), and Type IV laryngeal web (1). An antenatal diagnostic suspicion of duodenal atresia (obstruction) on ultrasound was described in two patients. UGI suggested a fenestrated duodenal web, visualized at ultrasound in two patients. Duodenal dilation was associated in two cases. The symptoms were feeding difficulties, nonbilious vomiting, upper abdominal distension, and poor growth. All presented with a pre-ampullary obstruction. Endoscopic confirmation was only possible in one patient. The older patient underwent an endoscopic resection of a duodenal web. In the other four, we performed a laparoscopic longitudinal antimesenteric duodenal incision, web resection (excision), and transverse suture (closure was performed) without duodenoplasty. Intraduodenal Indocyanine Green (ICG) visualization (under near-infrared light) was used in the last two cases. No postoperative complications were recorded, with a mean hospital stay of 8 days. A contrast study performed at 4 weeks demonstrated an improved proximal duodenal profile; patients tolerated a full diet and remained symptom-free. Conclusions: According to our experience with minimally invasive techniques, laparoscopy and endoscopy are effective and safe, supporting web resection for the management of a duodenal web without tapering of the proximal duodenum. They require advanced technical skills. Intraduodenal-ICG injection during laparoscopic treatment of Type 1 DA allows localization of the duodenal web, confirmation of bowel patency (bowel canalization) and the tightness of suture.

## 1. Introduction

Duodenal atresia (DA) represents a rare congenital malformation (anomaly) that occurs in approximately 1 per 5000 to 10,000 live births. Boys are affected more commonly than girls. Duodenal atresia is characterized by a complete obstruction of the lumen, while duodenal stenosis is characterized by an incomplete lumen obstruction. Usually, antenatally, with DA, which represents one of the most common causes of bowel obstruction, a polyhydramnios is visualized. It is localized more frequently (85–90%) in the second portion of the duodenum [1]. “There are several different theories that attempt to explain this pathology, such as vascular anomalies or abnormalities in neural cell migration”.

The most accredited consider DA as a consequence of duodenal re-canalization failure occurring between the eighth and the tenth week of embryological fetal development (Tandler’s Theory). The exact cause remains unknown.

DA is classified into three types: Type I (duodenal web/diaphragm), characterized by the presence of a mucosal diaphragmatic membrane with the integrity of duodenal muscular wall; Type II, in which the two pouches of the duodenum are connected by a short fibrous cord; and Type 3 (complete atresia), with a complete separation of the two ends of the duodenum [2].

In 30–52% of cases, it is isolated, while in the remaining cases it is associated with other congenital anomalies: Down’s Syndrome (trisomy 21), reported in approximately 20–30% of cases; as part of the VACTERL syndrome (with vertebral, anorectal, cardiac, esophageal atresia, renal, and limb anomalies); isolated cardiac anomalies, 25–30%; prematurity, 45%; growth retardation, 33%; other bowel anomalies, 25% [3]. Antenatally, the detection at ultrasound of the typical “double bubble” sign in the upper abdomen (in up to 44% of cases) allows the diagnosis. It represents the dilated fluid-filled stomach and proximal duodenum and is usually associate with polyhydramnios (32% to 81%) [3].

A duodenal web is a rarer cause of duodenal obstruction, which tends to cause a windsock deformity of the duodenal lumen. The incidence is reported to be between 1:10,000/20,000 and 1:40,000 [4,5]. Boys are more commonly affected than girls, although in some studies the sex distribution is the same. Webs are localized in the majority of cases in the second portion of the duodenum (85–90%). The third and fourth parts of the duodenum are involved in the 20% and 10%, respectively, occurring after Vater’s papilla and proximal to the ligament of Treitz [6,7].

The severity of symptoms, the age of onset, and radiological findings vary according to different factors: (a) the size and location of the web, (b) if the obstruction is total or partial, and (c) the size of the duodenal web’s fenestration. An incomplete web may present with intermittent symptoms that progress until obstruction is complete. Vomiting is the main symptom. Initially, patients may demonstrate tolerance to liquids, but not solids. In a later stage, patients may develop significant weight loss due to poor caloric intake. With persistent obstruction, patients may first develop progressive duodenal dilation followed by significant and progressive gastric dilatation with consequent loss of contractility of the stomach. Undigested food can accumulate, increasing the risk of pneumonia after aspiration. Therefore, a duodenal web, which causes a partial obstruction, could have a late presentation and a delayed and more challenging diagnosis.

Traditional repair of DA, including duodenal web, has been conducted via open duodeno–duodeno anastomosis with or without duodenoplasty via a transverse right supraumbilical laparotomic incision or by umbilical incision. 

MIS application has increased significantly in recent decades, in particular in neonatal surgery for correction of congenital anomalies as DA.

By a revision of literature, the first reported laparoscopic repairs were performed in 2001 by Bax [8] and in 2009 by Rothenberg [9]. In 2002, Gluer S. et al. described a case of laparoscopy reconstruction for an annular pancreas and malrotation [10], while only few published retrospective case series compared open with laparoscopic repair of congenital duodenal obstruction [3,11,12].

The aim of this study is to describe and analyze our mininvasive surgical strategy and management, detailing the aspects of laparoscopic technique. According to our experiences with neonatal mininvasive surgery in the treatment of other congenital malformations, we decided to evaluate patients with Type I DA who underwent laparoscopic treatment.

## 2. Material and Methods

### 2.1. Study Design and Population

Since 2009, at our Institution (Pediatric Surgery Unit of San Bortolo Hospital, Vicenza, Italy), we have treated duodenal atresia (DA) by minivasive surgery (laparoscopic or endoscopic approach). We retrospectively analyzed children who underwent mininvasive repair of Type I DA. We included all children with a late-presentation diagnosis of Type I DA treated by the same surgeon at our Department, from 2013 to 2023 and a follow-up period of at least 1 year. All patients who were treated with this method by the same surgeon since then were included in the study. Patients were selected among all the children affected with DA: we considered those who underwent mininvasive (endoscopic/laparoscopic) repair of Type I DA (fenestrated web) with a delayed presentation. We examined medical records analyzing and considering personal and perinatal data, associated anomalies, anthropometric data, associated gastrointestinal and respiratory symptoms, surgical technical details, complications and outcomes. Before the start of the study, all parents provided consent for the retrospective analysis of anonymized data for the purpose of clinical research.

#### Perinatal Data

Perinatal details such as sex, gestational age, birth weight, age at presentation, clinical presentation and associated anomalies were recorded for children with Type I DA. Associated anomalies included cardiovascular, gastrointestinal, anorectal, urogenital and skeletal malformations. We also considered radiological and intraoperative findings, surgical management and outcomes.

### 2.2. Inclusion and Exclusion Criteria

During the considered period, 15 patients affected by DA were treated at our Institution: 5 of them had a delayed clinical presentation and Type I DA characterized by a duodenal fenestrated web during diagnosis. One presented neonatal onset and was excluded by the study.

### 2.3. Investigations

Diagnostic examinations included ultrasound and upper-gastrointestinal study (UGI). Endoscopy was performed in all patients, but web confirmation was possible only in one case.

### 2.4. Surgical Procedures

#### 2.4.1. Endoscopic Resection

**Figure 1:**
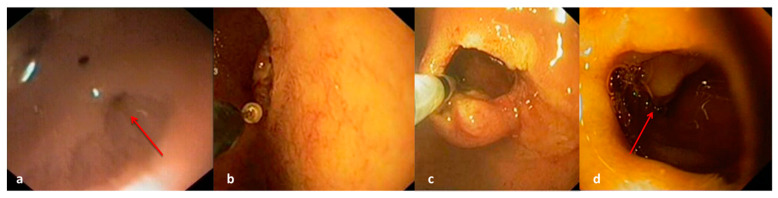
(**a**) Web: endoscopic view. Red arrow indicates web hole. (**b**) Electrosurgical knife with protected spherical tip. (**c**) Web resection. (**d**) Final view after web resection. Red arrow indicates Vater’s ampulla.

The first patient, the older one in our case series, underwent endoscopic web resection. Figure 1a. We used a flexible endoscope (GIF-Q180 series; Olympus America, Center Valley, PA, USA) and a disposable electrosurgical knife with a protected spherical ceramic tip, similar to an insulated tip (IT)-type knife (Olympus) (Figure 1b,c). The web appeared thin compared to the duodenal wall, making it possible to clearly distinguish the two. Localized the central fenestration, the electrosurgical knife was inserted and, applying energy, we proceeded toward the antimesenteric part of the web, ensuring a safe distance from the duodenal wall. 

The incision was gradually enlarged to reach a luminal calibre which allowed the transit of the endoscope. In this way, we were able to visualize Vater’s ampulla and to safely continue and complete the resection (Figure 1d). We avoided injecting any “protective” submucosal solutions in order to not reduce the endoluminal visibility and workspace [13].

#### 2.4.2. Laparoscopic Resection

**Figure 2:**
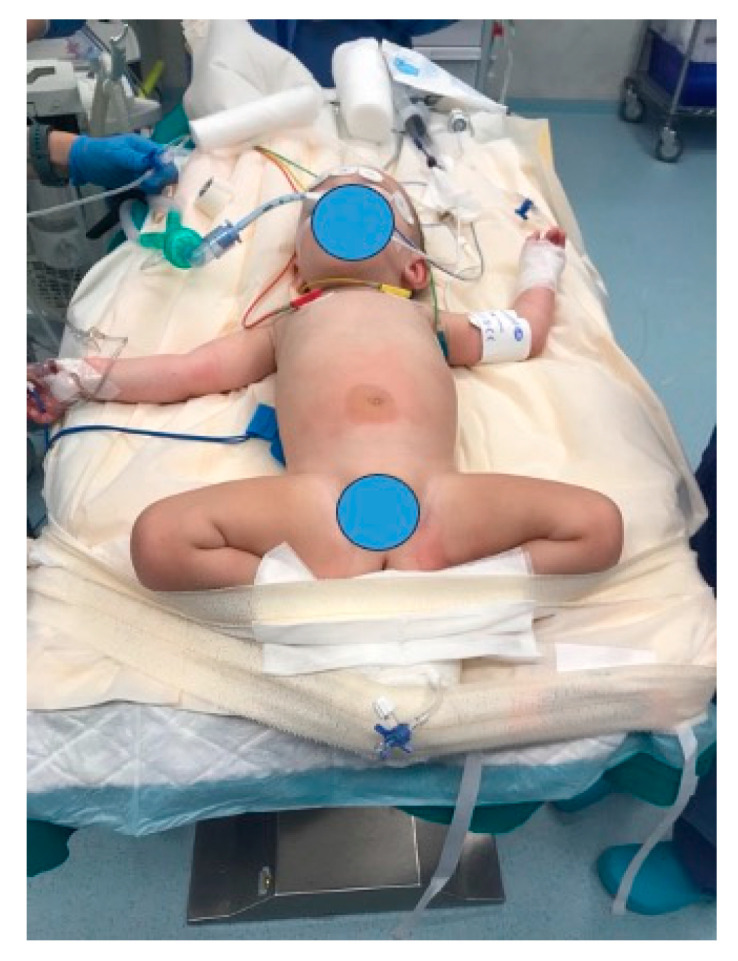
Patient position.

The patient is placed in the reverse Trendelenburg position at the lower edge of the operating table to provide convenient access to the operating field for the surgeon (Figure 2).

The procedure starts with open access umbilical incision. The umbilical artery and vein are visualized, dissected free, and ligated. Under direct vision, a 5 mm port is placed into the peritoneal cavity. A pneumoperitoneum with carbon dioxide (5–7 mmHg, 0.5 L/min) is generated and a 30° angle telescope is placed into the abdominal cavity, which is then explored to exclude additional anomalies (malrotation or intestinal atresia).

**Figure 3:**
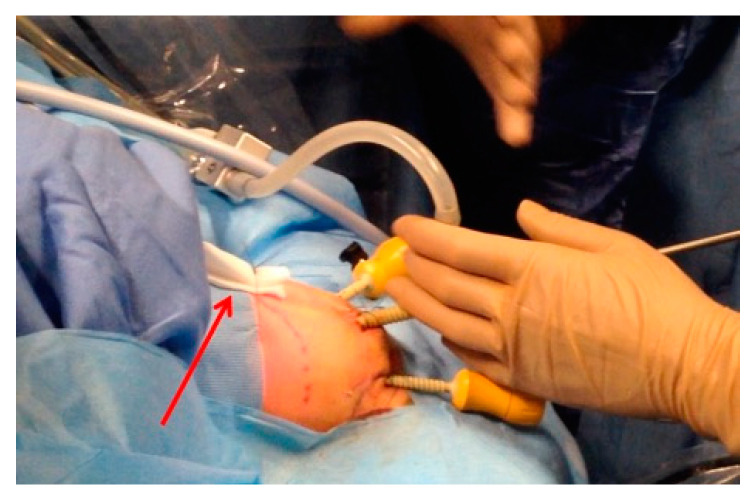
Rolled-up gauze (red arrow) for liver traction suture to avoid tissue laceration.

Two operative 3 mm trocars are then inserted under direct vision in the lower right and left quadrants. An additional 3 mm grasping forceps can be introduced in the epigastrium for lifting the liver. Our personal trick consists in positioning a one-stay percutaneous traction suture under the hepatic falciform ligament to lift up the liver, thus avoiding need for a third accessory port. The suture is usually tied over a rolled-up gauze to avoid tissue lacerations (Figure 3).

**Figure 4:**
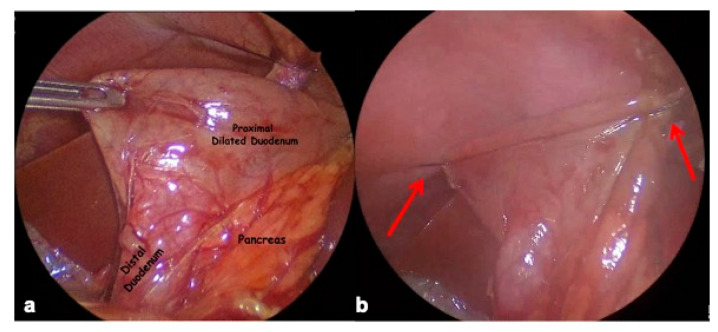
(**a**) Duodenum exposition: proximal dilated duodenum, transition zone with calliper reduction and normal duodenum; (**b**) traction of proximal dilated duodenum with two percutaneous sutures on left and right side (red arrows).

This maneuver improves visualization of the duodenum, especially its distal part. The gastrocolic ligament at the transverse colon is partially dissected from the stomach and duodenum and reflected inferiorly. Then, following the pylorus, the dilated proximal duodenal segment is identified and its mobilization from the retroperitoneal position is started. Dissection must be conducted with accuracy, staying on the anterolateral side of the duodenum to preserve the ampulla and pancreatic duct. This step was very challenging in the two patients with a huge dilatation and redundancy of the proximal duodenum. Another trick is to introduce a two-side stay suture transcutaneously in the upper quadrant of the abdomen through the right and left side of the dilated proximal duodenum (serosal layer) for traction. In this way, we correctly exposed the anterior surface of the dilated proximal duodenum, the transition zone with the calibre reduction and the normal distal duodenum (Figure 4a,b).

In accordance with the anatomy of the cases, the second and third duodenal portions could be adequately mobilized, using as much as possible a “no touch” technique to allow the repair.

**Figure 5:**
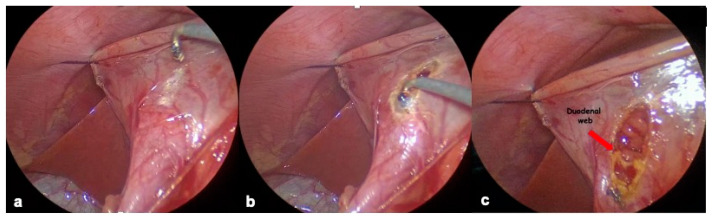
(**a**–**c**) Duodenotomy. (**a**) Incision site marked with monopolar hook; (**b**) opening duodenum; (**c**) duodenum opened over duodenal web (red arrow).

After correct exposition is reached, we performed an anterior duodenotomy, starting from the distal part of the dilated proximal duodenum, passing through the narrowed segment to reach the distal regular duodenum. We first mark the incision site with a monopolar hook; then, we open the duodenum with scissors positioned directly over the web (Figure 5a–c).

**Figure 6:**
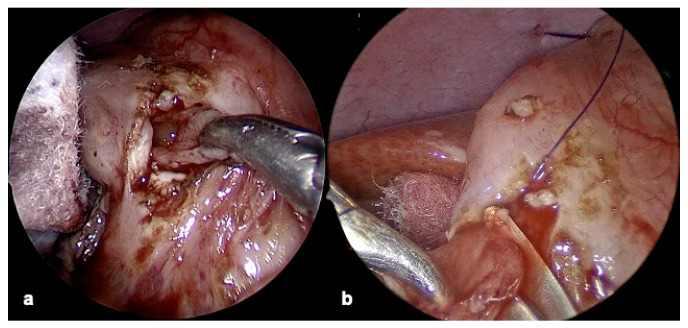
(**a**) Web exposition; (**b**) web excision.

Web resection starts from the central part and extends laterally toward the duodenal wall (anterolateral side). During the excision, the duodenotomy allows a clear visualization of Vater’s ampulla, which can be safely preserved (Figure 6a,b).

After web excision, we check the distal duodenum patency with a 4 Fr portion of the aspiration tube, which is introduced in the abdominal cavity through a trocar and then inside the duodenal lumen.

**Figure 7:**
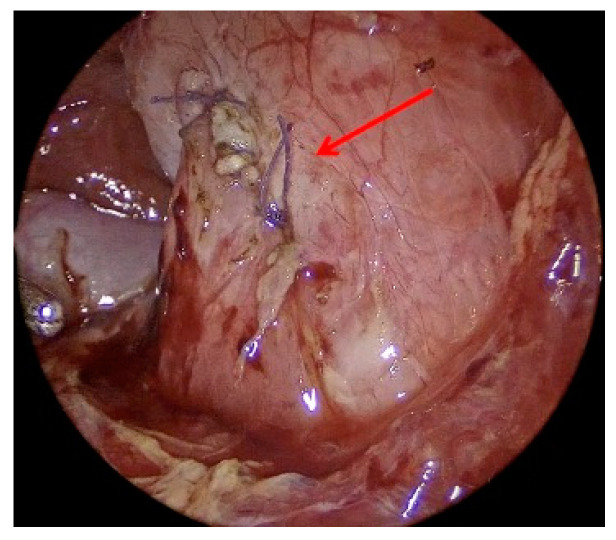
Duodenum closed transversely with 5/0 absorbable interrupted sutures (red arrow).

The longitudinal duodenotomy is then closed transversely in a single layer according to ‘Heineke-Mikulicz’ with 5/0 absorbable interrupted sutures (Figure 7).

An absorbable running suture could also be used. The type of suture (running or interrupted one) is chosen considering the laparoscopic space we have to perform the anastomose. We try to use the most ergonomic technique. The colon is then laid back over the duodenum and the trocars are removed under direct vision.

#### 2.4.3. Duodenal Web Localization

To better identify the position of the internal obstruction, it is possible to introduce and gently push down a nasogastric tube toward the proximal dilated duodenum. It puts the web under tension, clearly detecting an incisura on the duodenal surface. This maneuver is very simple during an open surgical approach but could be demanding during a laparoscopic procedure. It is not always possible when a huge dilation of the proximal duodenum is present. In these cases, we use Indocyanine Green (ICG): ICG is administrated through a nasogastric tube in the proximal dilated duodenum to identify the site of internal obstruction. 

**Figure 8:**
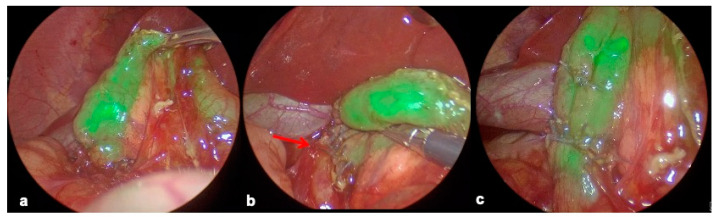
(**a**) Web localization by ICG; (**b**) suture (red arrow) check for potential leakage by ICG; (**c**) confirmation of bowel patency by ICG.

The site of the duodenal web was accurately identified using a laparoscopic system (KARL STORZ GmbH and Co. KG, Tuttlingen, Germany). The images are generated by a high-end full high-definition camera system (IMAGE1 S™ Rubina^®^, KARL STORZ, Tuttlingen, Germany) connected to a Telescope 30° 5 mm Karl Storz Endoscope which combines the latest 4K and fluorescence imaging technologies (NIR/ICG) (Figure 8a).

ICG helps to identify the exact site of where to perform the duodenotomy. This is especially useful for the windsock variant.

After duodenal web excision, the duodenotomy is closed in a transverse fashion. Another 5 mL of diluted ICG is then instilled to check the suture for potential leakage (Figure 8b) and to evaluate the bowel permeability **(**Figure 8c).

## 3. Results

A total of five children, born with Type I DA, were treated between 2013 and 2023 at the Pediatric Surgery Unit of San Bortolo Hospital: three boys and two girls with a median age of 5.5 months (range 3 to 11 months). Patient median weight was 4340 g (range 2800 g to 9800 g). An antenatal diagnostic suspicion of duodenal obstruction on ultrasound was only described in one patient, with a suspected double bubble image not confirmed at birth. Three patients underwent previous surgery for esophageal atresia (EA): two of them presented a long-gap Type A EA, requiring a gastrostomy immediately after birth and subsequent delayed primary anastomosis; one patient, born at 33 + 5 weeks’ of gestational age, presented a long-gap Type C EA. First, we performed gastrostomy to allow feeding and to postpone EA thoracoscopic repair when an adequate weight was reached. Multiple recorded associated anomalies were recorded in our patients, as described in Table 1.

**Figure 9:**
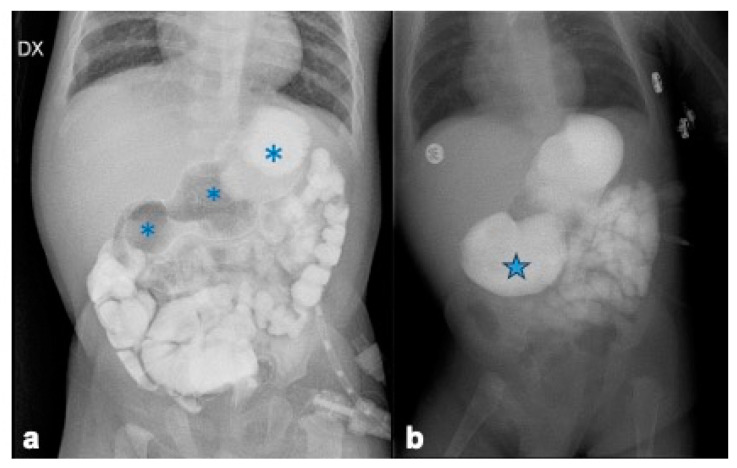
(**a**) Abnormally distended stomach and triple bubble sign (asterisk) consistent with diagnosis of congenital duodenal stenosis; (**b**) huge duodenal dilatation (star).

In the three patients affected by EA, Type I DA diagnosis was delayed after esophageal repair (respectively, 11 months, 6 months and 4 months of age). Initial enteral feeding via gastrostomy was poorly tolerated; after EA correction, oral feeding caused several episodes of upper-abdominal distension and nonbilious vomiting. It proved difficult to improve the patient’s nutrition, with subsequent poor growth. During the first month of life, the other two patients presented feeding difficulties associated with weight loss and recurrent episodes of non-bilious vomiting. At presentation, all patients were studied with plain abdominal X-ray, which detected a dilation of the first and second portion of the duodenum (a double bubble sign), as well as air in the distal bowel. Subsequently, we performed an upper-gastrointestinal study (UGI), showing a distended stomach associated with the dilated first and second duodenal portions (triple bubble signs), consistent with a diagnosis of congenital duodenal obstruction. In two patients, there was a huge duodenal dilation (Figure 9a,b).

**Figure 10:**
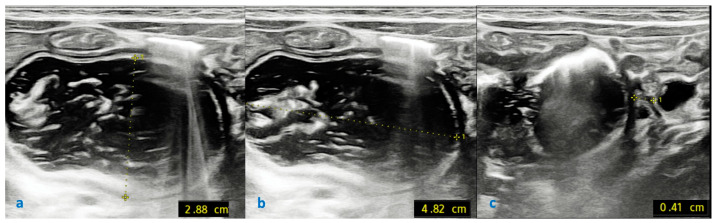
Ultrasound: dilated duodenum with transverse diameter of 2.88 cm (**a**) and longitudinal diameter of 4.82 cm (**b**); intraluminal diaphragm with central aperture of 0.41 cm at site of obstruction (**c**).

Abdominal ultrasound showed a markedly distended stomach and the water-refilling process of the duodenum, detecting a web between the second and third sections of the duodenum (Figure 10a–c).

According to our experiences in the mininvasive correction of congenital and acquired anomalies (thoracic, abdominal, urological) in newborns and infants and the use of ICG (administrated by systemic or endoluminal infusion), in two patients, we decided to inject a sterile ICG solution in a 8Fr naso-gastric tube to identify the site of internal duodenal obstruction. After exposing the duodenum, the site of the web was confirmed in two patients by the Rubina Telescope, which allowed us to visualize the cessation of indocyanine green fluorescence and its absence in the distal duodenum.

As reported by Esposito et al. [14], ICG dose commonly administered in Pediatric Surgery procedures ranges between 0.1 and 0.5 mg/kg, much lower than a toxic level. In most procedures, the administration is via intravenous injection, except for varicocele repair, in which the ICG is directly injected into the testis/dartos.

In our two patients, 25 mg of sterile indocyanine green was dissolved in 25 mL of distillated water (1 mg/mL concentration) to prepare the ICG solution; 10 mL of this solution was diluted 10 times to obtain a concentration of 0.1 mg/mL. The 5 mL of the ICG solution was inserted through an 8Fr nasogastric tube.

After duodenotomy, web resection and duodenoplasty, another 5 mL of the ICG solution was injected to check the suture for potential leakage and to evaluate the bowel permeability.

A nasogastric tube was left in place for two days after surgery. Patients started with oral liquid on the 3rd postoperative day and reached full oral feeding between the 5th and the 7th day after surgery. Bowel canalization was reached in three days. All four cases (except the first treated by endoscopy) were completed laparoscopically, and there were no intraoperative complications. The laparoscopic procedures were performed by the same senior surgeon, with no intraoperative complications and a conversion rate of zero. We had no postoperative leaks, no missed distal intestinal obstructions, and no short-term or long-term complications. Mean hospital length of stay was of 8 days. A contrast study was performed at 4 weeks after surgery, showing a reduction in proximal duodenum diameter and a regular duodenal transit; at follow up, all regular diet was well tolerated by all patients and they all are symptom-free.

## 4. Discussion

In this study, we described in detail the mininvasive treatment of Type I DA and the application of ICG as a novel trick for accurately detecting the precise site of the duodenal web, verifying the absence of anastomotic leakage and checking the adequate transit after duodenoplasty. 

The duodenal web was first reported by Boyd in 1845 [15]. It develops during fetal life, secondarily to a failure of the process of recanalization of the duodenal lumen (between the 8th and 10th week of gestation). Webs are usually located near the papilla; they are associated with other congenital anomalies (Trisomy 21, annular pancreas, anorectal malformation, cardiac defects, vertebral, malrotation) in more than 50% of patients. Diagnosis is usually antenatal for the presence of polyhydramnios and the double bubble signs, with the onset of symptoms at birth. With a fenestrated web, the presentation could be variable or delayed with signs and symptoms of partial bowel occlusions.

With breast feeding or artificial milk, a fenestrated web could be asymptomatic. As a child is weaned and started on solid foods, food elements could lead to web obstruction causing a subsequent dilation of the duodenum and stomach. The duodenum sometimes (as in two of our patients) becomes very dilated with kinking, atonic and with an ineffective peristalsis. More often, symptoms are (a) vomiting, bilious or not bilious, according to the level of the stenosis (in two-thirds of the cases, duodenal obstruction is distal to the ampulla, leading to bilious vomiting); (b) malnutrition/poor growth; (c) failure to thrive; (d) weight loss; (e) feeding difficulties associated (or not) to epigastric distension. Abdominal X-ray shows a typical distension of the stomach and duodenum with normal gas representation in the distal bowel. This condition is confirmed by upper-gastrointestinal study (that we have performed in all patients). According to Jangid et al., the retention of contrast in the duodenum for more than 6 h is indicative of web presence [16]. The size of fenestration is correlated to the severity of symptoms. If the lumen is only partially obstructed, the diagnosis could be delayed. Some reports describe a later onset in infancy or childhood [17,18].

Reviewing the literature, there are few studies or case reports describing a delayed presentation of a duodenal fenestrated web.

Compared to other types of DA (Type II and Type III) in which complete duodenal lumen obstruction clearly correlate with an antenatal diagnosis and a perinatal symptoms onset (usually at birth), in Type I DA, the presence of only a partial obstruction of the lumen can give a variety of presenting symptoms, causing a challenge. Velmishi et al. [2] reported a fenestrated web in a 16-year-old female patient. With a delayed presentation of months or especially of years, it is very difficult to consider a different diagnosis to a congenital cause. According to Tiwari et al. [19], the incidence of fenestrated webs ranges from 23.07% to 75% amongst all of the duodenal webs reported in the different studies collected. In our series, we have a delayed presentation in five out of six patients (83.3% of cases) ranging from 1 month to 11 months of life, probably due to the size of the fenestration (Table 2).

Usually, diagnosis can be established by plain abdominal radiography. The classical “double-bubble” appearance, pathognomonic of a complete duodenal atresia, may not be specific to fenestrated duodenal webs. Considering this type of DA, a radiological contrast study represents the gold standard procedure for diagnosis. Endoscopy could help in diagnosis, especially in older children, with duodenal dilatation and the presences of a fenestrated web, even if it is sometimes difficult to perform (as in two of our patients). In these cases, the diagnosis of the obstruction is indirect due to the dilation and kinking which make it impossible to explore the duodenum. Surgical options consist in duodenotomy with excision of the duodenal web or performance of a duodeno-duodenostomy or a duodenojejunostomy (bypassing the stenosis). The web excision by the open or laparoscopic approach is demanding because it may result in damage to the Vater’s papilla and/or biliary/pancreatic ducts that can open near or at the level of the web. More particularly, during laparoscopy, it could be difficult to localize the exact location of the web.

Considering the duodenal segment with a variation in calibre as the insertion site of the web could lead to the perform duodenotomy not in the right place. In wind-sock deformity, performing the duodenotomy at exactly this level in the duodenum could lead to not identify the web because it has a more proximal origin and prolapses distally.

Therefore, a combination of endoscopy and laparoscopy can be used to identify a web’s location, but sometimes this is not possible. Sometimes a nasogastric tube can be used to precisely identify a web’s location during operation by advancing it inside the duodenum, but this is not always possible. This is especially true with a huge dilation and kinking of the proximal duodenum. In our experience, we avoid the risk of an erroneous incision thanks to a correct exposition of the duodenum with traction sutures and the performance of a longitudinal anterolateral incision on the antimesenteric side starting from the dilated portion of duodenum over the calibre change, and extending distally for a short tract on the non-dilated duodenum. In this way, we were always able to identify the web.

The inability to pass a stiff catheter (the nasogastric tube or a small catheter introduced in the abdomen during laparoscopy) into the distal duodenum after duodenotomy should raise suspicion of a distal duodenal/jejunal obstruction, this demanding a careful inspection.

According to our great experience with the use of ICG in mininvasive neonatal and pediatric procedures (urological, abdominal, thoracic), in two patients presenting with a huge dilation, we decided to inject ICG through the nasogastric tube.

This maneuver allows us to correctly identify the position of the web by visualizing the green fluorescence in the proximal duodenum, and the lack of fluorescence in the distal duodenum. Once the web is excised and the duodenoplasty performed, we can use the same technique to evaluate the suture and to check for any leakage. Waiting few minutes with a gentle manipulation of the duodenum, we also can visualize progression of the dye throughout the whole duodenum and initial part of the jejunum.

This confirms the patency of the anastomosis and also allows evaluation of the presence of a second web, as suggested by Lee et al. [26]. Multiple DAs are rare, but many individual case reports have reported it [27,28]. Only 32 cases of double duodenal webs have been reported in the literature, two of which were reported in adults [23].

We have extensive and successful experience with the use of ICG in pyeloplasty. We inject it into the bladder to check the correct position of the JJ stent, visualizing the dye in the pelvis before completing the pyeloplasty. We have not recorded any complications. We are used to diluting ICG more than usual to reduce the dosage and so to avoid possible complications of toxicity from systemic absorption or from leakage into the abdominal cavity.

As reported by Ding et al., ICG is unstable and easily degraded under illumination, especially in acid conditions. In Type I DA with a fenestrated web, we administrated ICG in the stomach, where gastric acid increases its degradation, allowing, however, for the web to be identified [29,30].

“Considering fast degradation of ICG in acid conditions (as in the stomach) its administration must be performed at the right time, in close temporal proximity to what the Pediatric Surgeon wants to verify: the stop determined by the duodenal membrane or a potential leakage of suture or the patency of the bowel (distal duodenum and jejunum) [29,30].”

The main potential complications in the laparoscopic repair of DA can be the following: (a) anastomotic stricture; (b) anastomotic leakage; and (c) failure to verify distal bowel patency (third and fourth duodenal portions and jejunum). Correct positioning of the trocars is essential for the success of the procedure. Among the tips and tricks, the use of traction sutures on both the liver and the dilated proximal duodenum allows for excellent exposure of the duodenum, itself allowing the surgeon to freely use two hands to perform the suture. According to our experience, there are no differences between the interrupted suture or a continuous one. Some authors believe that interrupted suture is more effective in preventing stricture. We have used both sutures without postoperative stenosis or strictures. At the same time, we did not record any leakage. In the last two cases, ICG also helped in checking the anastomosis/suture, avoiding leakage, also allowing for the verification of the patency of the duodenum and jejunum. Moreover, laparoscopy allows the exploration of the whole bowel, in looking for other possible atretic tracts, as is usually performed in open surgery. Only a web (Type I atresia) could be missed, despite the reduced possibility of detecting distal atresia being extremely low (less than 2%). Another relevant advantage experienced with laparoscopic correction of DA is the faster bowel canalization. The reduced distal bowel manipulation, leads to a shorter ileus, an earlier resumption of oral feeding and a shorter time to full feeds, with a consequent reduction in postoperative hospitalization.

With the recent advancements in endoscopic therapy, including balloon dilation and endoscopic incisional therapy, endoluminal treatment for duodenal anomalies in children represents a possibility for reduced recovery time and avoiding potential postsurgical complications. Reports of endoluminal therapy for duodenal lesions in pediatric patients are growing and appear effective in the hands of experienced advanced Endoscopists [31].

However, there are limitations in performing a successful endoscopic recanalization of a duodenal web: (1) the size of the child in relation to the size of the instrument; (2) anatomical limitation: if the web is located more distally, near the Treitz’s ligament, this may create challenges to reach it with the scope; (3) the use of biopsy forceps or endoscopic sphincterotome with electrocautery increases the risk of duodenal perforation or treatment failure, above all in patients who have failed initial endoscopic management and require repeated dilations. These factors can greatly impact the success of endoscopic treatment [4,32]. Careful patients’ selection is critical in achieving successful endoscopic management in these patients. Sometimes, an intralesional corticosteroid infiltration at the site of the dilation is reported to help in decreasing the post-dilation inflammation and fibrosis and to achieve long-term luminal patency [31].

## 5. Conclusions

The laparoscopic repair of Type I DA with a fenestrated web represents a valid and successful technique. It is highly demanding and requires an adequate learning curve in neonatal procedures. The open approach is simpler compared to the laparoscopic one, given the possibility of direct bowel manipulation. However, the implementation of experience and the knowledge of specific technical tips and tricks allow to perform laparoscopic procedure safely and effectively. According to our experience, this approach reduces the risks deriving from bowel exteriorization, exposure, hydroelectrolytic losses and from bowel manipulation (adhesions). The use of ICG-guided surgery allows for a safe duodenal web identification, facilitating the web’s excision.

## Figures and Tables

**Table 1:** Data of our cohort.

	Sex	Age (Months)	Weight g	Associated Anomalies	Signs and Symptoms	Prenatal Findings
1	M	11	9800	Long-gap Type A EA, Type IV laryngeal web, hypospadia	Weight loss; feeding difficulties via gastrostomy; nonbilious vomiting; abdominal distension	No
2	F	3	2800	Long-gap Type A EA, Ladd’s band, malrotation, cloaca, VUR	Weight loss; feeding difficulties via gastrostomy; nonbilious vomiting; abdominal distension	No
3	M	3	2900	Pyelectasis, accessory spleen	Weight loss; feeding difficulties via gastrostomy; nonbilious vomiting	Yes
4	F	4	3050	Pyelectasis	Weight loss; feeding difficulties via gastrostomy; nonbilious vomiting	No
5	M	6	3200	Long-gap Type C EA, hexadactyly, anorectal malformation (anterior ectopic anus), VUR	Weight loss; feeding difficulties via gastrostomy; nonbilious vomiting; abdominal distension	No

**Table 2:** Review of literature of various studies with duodenal atresia, duodenal webs and delayed presentation.

Study by Authors	Year Published	Total n° of Patients with DA	Total n° of Patients with Fenestrated Duodenal Web	Total Number of Patients with Delayed Presentation
Krieg et al. [20]	1937	18	3	3
Nawaz et al. [21]	2004	8	6	2
Kaddah et al. [22]	2006	12	7	7
Sarin et al. [23]	2012	18	3	3
Lin et al. [24]	2012	13	7	7
S. Kumar et al. [25]	2015	13	3	3
Mousavi et al. [6]	2016	19	8	7
Tiwari C. et al. [19]	2023	/	6	6
Chiarenza et al. [current paper]	2025	15	6	5

## Data Availability

Data are available on request from the corresponding author.

## Abbreviations

DA	Duodenal Atresia
EA	Esophageal Atresia
VACTERL	Vertebral, Anorectal, Cardiac, Esophageal Atresia, Renal and Limb Anomalies
MIS	Mininimally Invasive Surgery
ICG	Indocyanine Green
NIR	Near-Infrared
VUR	Vesical Ureteral Reflux
UGI	Upper-Gastrointestinal Study
US	Ultrasound
